# Advanced practice nursing: Should research be the icing on the cake?

**DOI:** 10.1002/nop2.1122

**Published:** 2021-10-30

**Authors:** Mary Casey, Laserina O’ Connor

**Affiliations:** ^1^ College of Health and Agricultural Sciences UCD School of Nursing, Midwifery and Health Systems Dublin 4 UK

Advanced practice nursing is defined as a career pathway for registered nurses, committed to continuing professional development and clinical supervision, to practice at a higher level of capability as independent, autonomous and expert practitioners. The advanced practice role requires the application of relevant research and management, knowledge and skills in order to provide high‐quality patient outcomes and ensure implementation of evidence‐based practice. This knowledge and skill set is required to effectively perform their role but may not be formally recognized as an important skill set and may account for the finding that there is a reduced level of interest in becoming advanced practitioners (Woo et al., 2020). Ironically, the World Health Organization's global nursing strategy mentions evidence‐based practice, but competence in “evidence generation” is deemed relevant only to nursing leaders (WHO, 2016).

Current health policy in Ireland requires a reorientation of the health services towards primary and social care in the community “delivered at the lowest appropriate level of complexity through a health service that is well organised and managed to enable comprehensive care pathways that patients can easily access, and service providers can easily deliver” (Department of Health, 2017:20). Likewise, the Health Service Executive developed National Clinical and Integrated Care Programmes as priority programmes to design, develop and progressively implement models of care that incorporate cross‐service, multidisciplinary care and supports which facilitate the delivery of high‐quality evidence‐based and coordinated care. Therefore, a reorganization of the boundaries between secondary care and tertiary care to move services traditionally provided by hospitals to community‐based providers must be achieved in a post‐COVID‐19 environment where greater emphasis will be on the community care context. We suggest that Advanced Nurse Practitioners (ANPs) are best positioned to undertake these boundary spanning roles and they are, in some cases, currently undertaking these roles. They are working at very senior organizational levels acting as practice leaders, managing their own case workload and engaging across professional, organizational, agency and system boundaries to improve services and develop practice. Is this an unfair burden or a realistic expectation to place on ANPs?

Based on our clinical and academic experiences, we note that engagement in research and clinical practice makes competing demands on the ANPs time and expertise, and can be a barrier to motivation. To date, it is the clinical role that is more emphasized in the health service. This unfortunately restricts the ANP’s capacity for personal and professional growth and limits opportunities for capacity building. Real‐life engagement in research activity is essential and while learning about research and leadership from a theoretical perspective is essential, practical application in a safe learning environment is required to ensure that that the ANP is fully enabled to work within his/her full scope of practice to deliver, organize, manage and evaluate essential comprehensive care pathways that patients can easily access.

Some 10 years ago, the Royal College of Surgeons in Ireland (2010) highlighted that the research element of the ANP role was the least developed, possibly due to the lack of time to engage in research. Indeed, Casey et al. (2015) found that only a small minority of ANPs suggested that engaging in independent research was achievable or seen as an important element of the advanced nursing practice role. This continues to be our tangible experience and although there are an array of interpretations as to what “participating in research” actually consists of, engagement in research activity is still undervalued and under‐resourced in nursing (Gibson, 2019). Clinical leadership as a component of educational engagement is also underdeveloped (Elliott & Walden, 2014). While the educational preparation for these roles nationally has been improved, it is our experience that current ANP candidates as well as existing registered advanced nurse practitioners (RANP) continue to experience a lack of confidence in exercising competency in these two aspects of their roles. This needs to be urgently addressed in order to ensure that current ANP candidates are fit for purpose, fit for practice and fit for award and that existing RANPs have a pathway for ongoing professional development, engagement in scholarly activity and service improvement.

Although the advanced practice nursing role primarily focuses on delivering the clinical aspects of patient care, the other pillars of advanced practice (research, education and leadership/management) should not be overlooked as they are key to role actualization. While leadership as a competency is identified and indeed threaded through the competencies, it is within the knowledge domain (Domain 3) that the main emphasis on research is located. In this domain, it requires that the ANP candidate actively contributes to the professional body of knowledge related to the ANP’s area of advanced practice (Nursing & Midwifery Board of Ireland, 2017). We know that activities associated with leadership and research are two areas which remain a challenge for ANPs in terms of leading practice change. Could the fact that “research,” as an distinct activity, is somewhat obfuscated within these competency domains be directly contributing to its poor understanding and engagement?

Enhanced educational preparation in relation to attainment of leadership competency and research knowledge, attitude and skills involving real‐life experience under close academic supervision is essential. This will improve ANP candidate's level of confidence and experience both in engaging with leadership activities and in conducting research. The two activities could be meaningfully integrated whereby the leadership activities could include engaging in group work to identify a shared clinical problem and providing a rationale for a change in practice. Similarly, the research learning activities could include co‐creation of a research proposal, application for ethical approval to undertake a pilot research project within his/her own organization and then undertaking this research. A pilot study would be sufficient, and this provides a “win win” for all concerned—where the student has enhanced leadership and research learning and the actual clinical site receives a pilot tested albeit small improvement in practice. It is possible that a range of pilot projects could be identified by the Director of Nursing, and in this way, the current bureaucracy associated with securing ethical approval could be significantly reduced and this also reduces unnecessary delays which cause increased anxiety levels among the ANP candidates. With careful planning, it is possible for a number of ANP candidates to be able to engage with different aspects of the same study topic under the expert supervision and creativity of the academic supervisor. Opportunities for co‐supervision could also be created, thereby enhancing the mentorship role of an RANP in relation to research. A cleverly constructed doctorate programme in nursing could consider such engagement in mentorship activities as part learning towards completing a level 10 doctorate module. A direct advantage of building these types of relationships is greater unity within the profession and an increased understanding of both the clinical and academic worlds of professional nursing. Increased job satisfaction would result, and recruitment and retention of nurses to future roles in both clinical and academic nursing would be more attractive and sustainable.

From a global perspective, nurses practice at varying degrees of expertise and work within a range of different contexts and specialities. Advanced practice roles are one of the largest expanding workforces. According to the Chief Nurse in Ireland, “having advanced practitioners at the point of care delivery will not only support the delivery of integrated care across our health services, it will be a key enabler for the delivery of Sláintecare” (Department of Health, 2019:6). As such, healthcare policymakers and administrators capitalize on the variability and versatility of nursing practice to meet escalating healthcare demands through new models of care. Indeed, one could also argue that since COVID‐19 a comprehensive role evaluation process would be helpful as potentially, there is a large number of people that are already, or could be working at, advanced clinical practice level. Just imagine the value‐added and the cost‐saving benefits of enactment of these ANP roles in the long term. The time is ripe, in a post‐COVID‐19 context, to demonstrate our commitment to these roles and to insist on the provision of protected scholarly time for advanced nurse practitioner as an essential part of their employment contract.

We need to outline a pathway for national recognition of these complex high‐level roles against an agreed set of academic and clinical standards. This is about providing opportunities for RANPs to undertake:

- a range of level 10 academic modules that accumulatively can lead to an academic qualification at Professional Doctorate level.
- a comprehensive set of strategies for broader types of engagement in nursing research, such as implementation research and action research projects contributing to the nursing research agenda.
- more public visibility of their leadership roles in both clinical and organizational contexts.

These activities must be developed in parallel with support for those who wish to undertake doctoral and postdoctoral research. In a post‐COVID‐19 environment, there is a need for nursing education to explore new ways of doing things. It is timely to again consider the evolution of the professional doctorate to help move the profession forward. We need to aim for the next generation of nurses to be taught by highly qualified clinicians who have excellent leadership and teaching skills and are also active researchers. For now, we need to develop a culture where research is a transformative ingredient rather than the “icing on the cake.”

## CONFLICT OF INTEREST

Both authors confirm there are no other conflicts of interest to both authors.

## AUTHOR CONTRIBUTIONS

Both authors have contributed equally and meet the criteria for authorship and have approved the final version.

## ETHICAL APPROVAL

Ethical approval is not required for this editorial.

## Data Availability

Data sharing is not applicable—no new data generated.
